# Diaphragmatic Amyloidosis Causing Respiratory Failure: A Case Report and Review of Literature

**DOI:** 10.1155/2015/917157

**Published:** 2015-10-26

**Authors:** Aleksey Novikov, Horatio Holzer, Robert A. DeSimone, Ghaith Abu-Zeinah, David J. Pisapia, Tomer M. Mark, Raymond D. Pastore

**Affiliations:** ^1^Department of Internal Medicine, New York Presbyterian Hospital, Weill Cornell Medical College, New York, NY 10065-4897, USA; ^2^Department of Pathology and Laboratory Medicine, New York Presbyterian Hospital, Weill Cornell Medical College, New York, NY 10065-4897, USA

## Abstract

Neuromuscular respiratory failure is a rare complication of systemic immunoglobulin light chain amyloidosis. We describe a case of a 70-year-old Caucasian man with multiple myeloma who presented with worsening dyspnea. The patient was diagnosed with and treated for congestive heart failure but continued to suffer from hypercapnic respiratory insufficiency. He had restrictive physiology on pulmonary function tests and abnormal phrenic nerve conduction studies, consistent with neuromuscular respiratory failure. The diagnosis of systemic immunoglobulin light chain amyloidosis was made based on the clinical context and a cardiac biopsy. Despite treatment attempts, the patient passed away in the intensive care unit from hypercapnic respiratory failure. Autopsy revealed dense diaphragmatic amyloid deposits without phrenic nerve infiltration or demyelination or lung parenchymal involvement. Only 5 cases of neuromuscular respiratory failure due to amyloid infiltration of the diaphragm have been described. All cases, including this, were characterized by rapid progression and high mortality. Therefore, diaphragmatic amyloidosis should be on the differential for progressive neuromuscular respiratory failure in patients with multiple myeloma or any other monoclonal gammopathy. Given its poor prognosis, early recognition of this condition is essential in order to address goals of care and encourage pursuit of palliative measures.

## 1. Case Report

A 70-year-old Caucasian man with a previously diagnosed, IgG-kappa multiple myeloma presented with a 3-month history of worsening dyspnea, dysphagia, and weight loss. The diagnosis of multiple myeloma was established one year prior to presentation with a serum kappa free light chain (K-FLC) level of 685 mg/dL and a lambda FLC level of 0.13 mg/dL (kappa/lambda (K/L) ratio of 5269). The patient had no lytic bone lesions or liver or heart involvement and had a normocellular bone marrow with 70% plasma cells. He was never noted to have macroglossia or albuminuria. His past medical history was otherwise notable for hypertension, type II diabetes mellitus, atrial fibrillation, and chronic kidney disease with Bence Jones proteinuria (K-FLC proteinuria of 691 mg/dL). Upon initial evaluation, the patient was hypoxic to 78% in room air. He appeared tachypneic, with an increased work of breathing and accessory muscle use. Auscultation revealed overall reduced breath sounds, bibasilar crackles, and an irregular cardiac rhythm with a holosystolic grade III/VI murmur loudest at the cardiac apex.

The patient's arterial blood gas revealed a pH of 7.31, a pCO_2_ of 60, and a pO_2_ of 60 mmHg. His labs otherwise revealed an bicarbonate of 33 mmol/L, a creatinine of 2.55 mg/dL (increased from a baseline of 1.2 mg/dL), a hemoglobin of 10.3 g/dL, and a white blood cell count of 7.4 × 10^3^ per *μ*L. The patient also had a B-type natriuretic peptide level of 572 pg/mL and serial cardiac troponin I levels of 0.22, 0.22, and 0.21 ng/mL drawn 6 to 8 hours apart. His K-FLC level was 1379 mg/dL with L-FLC of 0.65 mg/dL (K/L-FLC ratio of 2121).

A chest radiograph showed mild pulmonary vascular congestion with small bilateral pleural effusions. His echocardiogram was notable for elevated pulmonary artery systolic pressure of 64 mmHg, biatrial dilatation with normal sized ventricles, and a preserved ejection fraction. A ventilation-perfusion scan was negative for acute or chronic pulmonary embolus.

Subsequently, the patient was admitted to the cardiac stepdown unit for inotropic diuresis and noninvasive positive pressure ventilation (NIPPV). After diuresis, a right heart catheterization (RHC) revealed PA pressure of 34/9/18 mmHg (systolic/diastolic/mean) and a pulmonary capillary wedge pressure of 10 mmHg.

Despite effective diuresis, the patient went into a worsening hypercapnic respiratory failure with almost continuous dependence on NIPPV. His pulmonary function tests revealed restrictive physiology with FEV1 of 61%, FVC of 58%, and FEV/FVC of 104%. A subsequent nerve conduction study was suggestive of bilateral phrenic axonal neuropathy.

With his worsening dysphagia and respiratory muscle weakness, the course was further complicated by aspiration pneumonitis. A barium swallow esophagram showed narrowing of the gastroesophageal junction and tertiary contractions in the esophagus. An upper endoscopy was unrevealing and biopsy samples were negative for amyloidosis, confirmed by lack of Congo red staining (not shown). A fat pad biopsy was also negative for amyloid.

Due to deteriorating cardiopulmonary and renal status, the patient was transferred to our cardiac intensive care unit. A repeat RHC was performed and a right ventricular biopsy was diagnostic of cardiac amyloidosis. The patient subsequently received 3 doses of bortezomib and dexamethasone but passed away soon after from the multiorgan system failure, approximately 5 weeks after admission.

**Figure 1:**
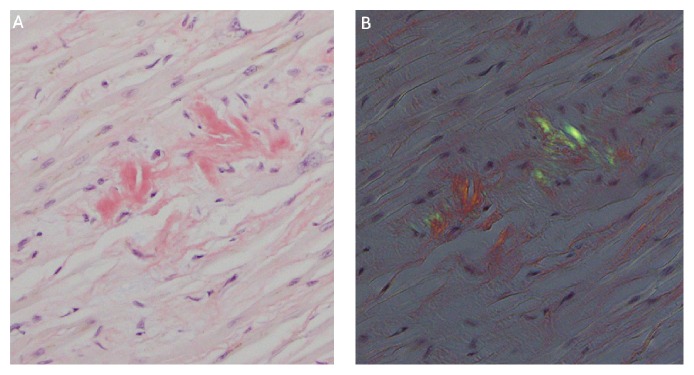
Cardiac amyloidosis. (A) Congo red stain of left ventricle showing focal positive staining (red-orange) of amorphous extracellular material, 400x. (B) Congo red stain of left ventricle showing apple-green birefringence under polarized light, diagnostic of amyloid, 400x.

**Figure 2:**
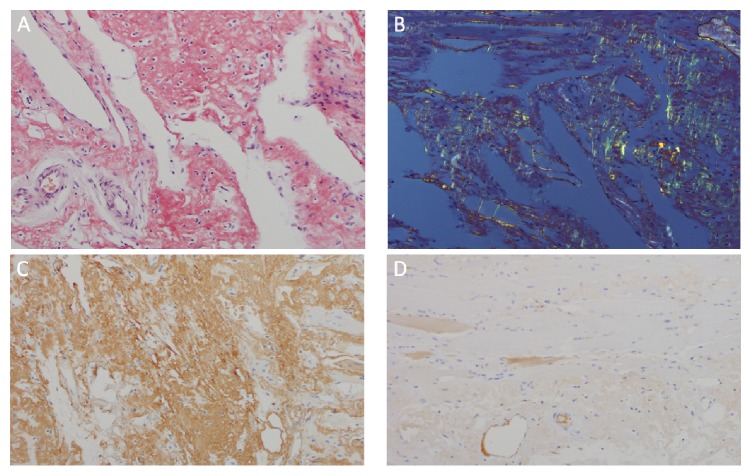
Diaphragmatic amyloidosis. (A) Congo red stain of diaphragm showing diffuse positive staining (red-orange) of amorphous extracellular material, 200x. (B) Congo red stain of diaphragm showing apple-green birefringence under polarized light, 200x. (C) Kappa light chain immunostain of diaphragm showing diffuse positivity, diagnostic of kappa amyloid light chain amyloidosis, 20x. (D) Negative lambda light chain immunostain of diaphragm, 20x.

**Figure 3:**
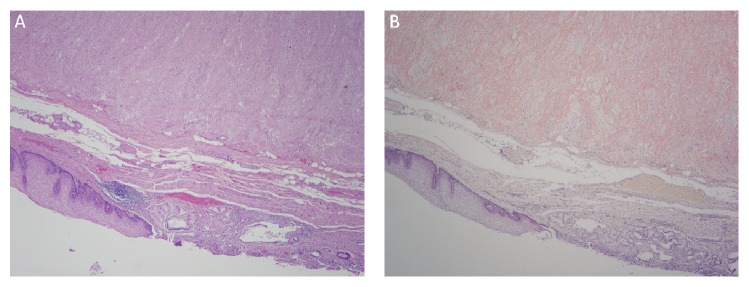
Gastroesophageal junction amyloidosis. (A) Hematoxylin and eosin stain of gastroesophageal junction showing a thickened muscularis propria, 20x. (B) Congo red stain showing diffuse extracellular positive staining (red-orange) restricted to the muscularis propria, 20x.

**Figure 4:**
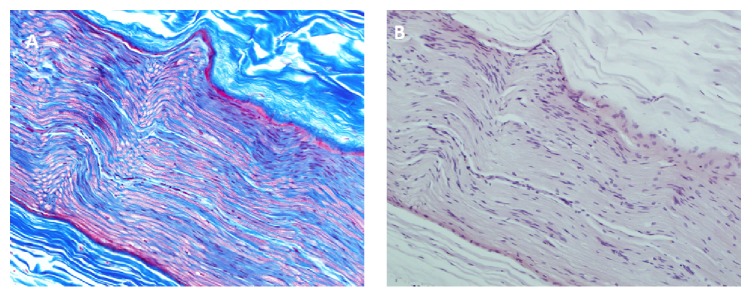
Phrenic nerve. (A) Trichrome staining of right phrenic nerve showing preservation of myelin sheath, 400x. (B) Congo red stain of right phrenic nerve, negative for amyloidosis, 400x.

Autopsy revealed IgG-kappa amyloid deposits in the left and right ventricles (Figure 1), the diaphragm (Figure 2), and the gastroesophageal junction (Figure 3). There was no demyelination, amyloid deposition, or other abnormalities of the phrenic nerves (Figure 4), nor was there amyloid infiltration of the lung parenchyma.

## 2. Discussion

The differential diagnosis of hypercapnic respiratory failure is extremely broad. It is ultimately the result of inadequate ventilation, which leads to an increase of the partial pressure of carbon dioxide in the blood. This subsequently leads to acidemia and concurrent hyperkalemia, both of which promote arrhythmias, muscle weakness, and CNS depression leading to death. This failure can be caused by disease in any organ system that is essential to the initiation and propagation of breathing including the airways, alveoli, central nervous system, peripheral nervous system, respiratory muscles including diaphragm, and chest wall. We propose that in this case the failure was caused by a mechanical defect in diaphragmatic function secondary to light chain amyloid infiltration of the diaphragm. We demonstrate sparing of the phrenic nerve and lung parenchyma on pathology, further supporting the hypothesis that diaphragmatic infiltration is the primary cause of respiratory failure in this reported case.

This rapidly progressive hypercapnic respiratory failure secondary to infiltration of the diaphragm by amyloid is only the fifth such case described in the literature. We have conducted an extensive search using National Library of Medicine database, looking for the words “amyloid”, “amyloidosis”, and “diaphragm”. We were able to find only 4 other relevant cases in the literature. The first published article describes a 56-year-old black man with multiple myeloma [1]. Autopsy from this case showed amyloid infiltration of the diaphragm, without mention of amyloid infiltration of the phrenic nerves or lung parenchyma. Two other articles describe single cases of a 55-year-old man and a 73-year-old woman with a primary hypercapnic respiratory failure, both found to have amyloid infiltration of the diaphragm with sparing of the lungs and phrenic nerves [2, 3]. Postmortem investigation suggested the pathophysiology of hypercapnic respiratory failure was attributable to amyloid infiltration of the diaphragm, in the absence of lung and phrenic nerve involvement.

To our knowledge, there is only one case report that proposes an alternative theory of respiratory failure driven by a peripheral nerve infiltration with amyloid [4]. This conclusion is based on the results of the abnormal neural conduction studies and fluoroscopy and spirometry studies. It is important to note that this report does not demonstrate a primary pathology of the phrenic nerves. On the other hand, autopsy results from all the other studies have shown amyloid infiltration only in the diaphragm, supporting the notion that diaphragmatic amyloidosis is the more likely etiology of respiratory failure.

Both our case and that by Berk et al. show abnormal nerve conduction studies. However, in the absence of pathology to suggest nervous system involvement or demyelination, it is very unlikely for phrenic nerve neuropathy to be the cause of respiratory failure in such patients. Therefore, nerve conduction studies should not be considered diagnostic. This becomes important when measures such as diaphragmatic pacing are considered, as it may be of benefit when the nervous system is involved but unlikely to benefit a diaphragm infiltrated by amyloid.

This case report highlights a rare but a rapidly fatal complication of systemic amyloidosis. The reported cases over the past 30 years show a 100% association between amyloid infiltration of the diaphragm and respiratory failure. In 4 out of the 5 cases of diaphragmatic amyloidosis reviewed, the diagnosis of respiratory failure carried an 80% mortality rate within 1 month. Only in 1 case did the patient survive hospitalization [4]. Given its high case mortality, findings of progressive respiratory failure in the setting of systemic amyloidosis should trigger goals of care discussions. While we have found no evidence to support the notion that amyloidosis of the phrenic nerves causes respiratory failure, we do think that the amyloid-induced respiratory failure should continue to be studied as diaphragmatic pacing could be considered in certain cases of phrenic nerve damage.
